# A qualitative study on health workers’ and community members’ perceived sources, role of information and communication on malaria treatment, prevention and control in southeast Nigeria

**DOI:** 10.1186/s12879-015-1187-2

**Published:** 2015-10-22

**Authors:** Jane C Umeano-Enemuoh, Benjamim Uzochukwu, Nkoli Ezumah, Lindsay Mangham-Jefferies, Virginia Wiseman, Obinna Onwujekwe

**Affiliations:** Department of Community Medicine, University of Nigeria, Enugu-Campus, Enugu, Nigeria; Health Policy Research Group, Department of Pharmacology and Therapeutics, University of Nigeria, Enugu Campus,, Enugu, Nigeria; Department of Health Adminstration and Management, University of Nigeria, Enugu Campus, Enugu, Nigeria; Department of Sociology/Anthropology, University of Nigeria, Nsukka, Nigeria; Department of Global Health and Development, London School of Hygiene and Tropical Medicine, London, UK; School of Public Health and Community Medicine, University of New South Wales, Sydney, Australia

## Abstract

**Background:**

It has been widely acknowledged that well-planned and executed communication programmes can contribute to achieving malaria prevention and treatment goals. This however requires a good understanding of current sources and roles of information used by both health workers and communities. The study aimed at determining health workers’ and community members’ sources, value and use of information on malaria prevention and treatment in Nigeria.

**Methods:**

Qualitative data was collected from six selected communities (three urban and three rural) in Enugu state, southeast Nigeria. A total of 18 Focus Group Discussions (FGDs) with 179 community members and 26 in-depth interviews (IDIs) with health workers in public and private health facilities were used to collect data on where people receive treatment for malaria and access information on malaria. The FGDS and IDIs also provided data on the values, uses and effects of information and communication on malaria treatment seeking and provision of services.

**Results:**

The findings revealed that the major sources of information on malaria for health workers and community members were advertisements in the mass media, workshops and seminars organized by donor agencies, facility supervision, posters, other health workers, television and radio adverts. Community involvement in the design and delivery of information on malaria control was seen as a strong strategy for improving both consumer and provider knowledge. Information from the different sources catalyzed appropriate provision and consumption of malaria treatment amongst health workers and community members.

**Conclusion:**

Health workers and consumers receive information on malaria prevention and treatment from multiple sources of communication and information, which they find useful. Harnessing these information sources to encourage consistent and accurate messages around malaria prevention and treatment is a necessary first step in the design and implementation of malaria communication and behaviour change interventions and ultimately for the sustained control of malaria.

## Background

Malaria is a major public health problem in many sub-Saharan African countries [1–3]. It mostly affects children under-five years of age, pregnant women and migrants/visitors from non-malaria endemic regions [4]. In general, malaria is responsible for the high rate of maternal health burden, absenteeism among school children, impoverishment, reduction in work productivity and capacity. About 40 % of the world’s population are affected by malaria and its burden is most prevalent amongst people of low socio-economic status [2, 5].

Malaria is a major cause of illness and death among adults and children in Nigeria [6, 7]. The prevalence of malaria, especially amongst children has been reported to be high in Nigeria at around 43.2 % [6]. Up to half of Nigeria’s population is at risk of at least one malaria episode every year [7]. The disease accounts for 60 % of out-patient visits and 30 % of hospitalizations among children under five, 25 % deaths of infants and 11 % maternal mortality [4]. The occurrence of malaria in some households causes significant economic burden which often leads to catastrophic health expenditures [8].

Key malaria prevention tools in Nigeria include prevention through the distribution of long lasting insecticide-treated nets (LLINs) and indoor residual spraying [9]. The distribution of LLINs is primarily through free public sector campaigns such as integration with immunization and antenatal care services. Some LLINs are subsidized through the commercial sector [9]. The first line drugs are artemisinin-based combination therapies (ACTs), which should only be taken after parasitological diagnosis with either microscopy or rapid diagnostic tests (RDTs). Sulphadoxine pyrimethamine (SP) is used for intermittent preventive treatment in pregnancy.

The levels of coverage with these different malaria control tools remains low in Nigeria [10]. A range of explanations have been put forward as to why this is the case. Several studies point to low levels of consumer and provider knowledge and awareness of the availability and usefulness of the various malaria control tools [3, 11]. Deficiencies in the practices of both private and public providers also compromise the effectiveness and cost-effectiveness of malaria-case management and have led to calls for a variety of interventions to improve the quality of treatment and advice given [3, 12].

A recent survey from Nigeria showed that few febrile patients attending public health facilities, pharmacies and patent medicine dealers received recommended treatment for malaria and those that did were frequently given an incorrect dose [3]. The same study also reported that it was common for febrile patients to request a specific antimalarial and in most cases, this was not the nationally recommended treatment [3]. Another Nigerian study showed that patients commonly misinterpret signs and symptoms of malaria due to their personal experience or mis-information received from friends, relations and neighbors [13]. In Nigeria, there is mounting evidence that malaria symptoms are often perceived wrongly or not recognized and continue to go untreated [13, 14].

Effective communication helps to ensure that malaria control tools are appropriately delivered and consumed [15, 16]. Several studies in Nigeria and in other malaria endemic countries have shown the positive effect of using structured education initiatives or programmes on increasing coverage, improving treatment and increasing knowledge of providers and community members [17–19]. A study in Tanzania showed that 3 million people were reached with information on malaria prevention and treatment from road shows and mobile video units [20]. Studies from other African countries show that information delivered through print media, health workers, posters, radio and television on malaria prevention and treatment improved malaria health seeking and treatment behaviors as well as malaria prevention [17, 18, 21]. Studies also show that it is important to ensure that information given to both providers and consumers is consistent and accurate [22].

It is widely acknowledged that communication is a vital tool in malaria programmes [23]. The communication strategic framework developed by the Federal Ministry of Health in Nigeria in 2008 is designed to raise awareness for malaria prevention and control strategies [1]. The strategy recommends the use of mass media, community mobilization, advocacy and other forms of communication to improve the knowledge, attitude and practices on malaria [1]. The practical design and implementation of these strategies however needs to be informed by an understanding of the different sources of information and types of communication channels currently used in the local context [7, 15, 16].

There presently exists a paucity of information on the different sources of information on malaria and how these sources affect the provision and use of malaria preventive and treatment tools. Societal and provider perceptions of the usefulness of this information are also not well understood. This paper provides new information on health worker and community perceptions of the importance, uses and roles of information and communication for improving malaria prevention and treatment in southeast, Nigeria. It also helps to support the future development of malaria communication strategies to ensure they are context appropriate and evidence-based.

## Methodology

### Study area

The study was undertaken in Enugu state, south east Nigeria in November, 2010. Two sites, Enugu (urban) and Udi (rural) communities were selected and used for the study in order to reflect broad perceptions on the use of information and communication for malaria prevention and control. The study sites are similar in terms of language and culture but differ in the number of health facilities. Due to the rural nature of Udi LGA, it has fewer public and private health facilities while the reverse is the case in Enugu.

Enugu state is geographically located in the southern zone of Nigeria between 7°10’N and 7°45’N of Equator and on longitude of 7.4878°E and latitude of 6.4231°N. The bioclimatic zone is rainforest in nature with annual rainfall between 152 cm and 203 cm and temperature ranges from 22.2 °C-30.6 °C. The state has a land area of 7,617.82 square kilometres and a population of 3,289,589 people. The activities of the majority of the population include farming, fishing, wine tapping, and poultry keeping and rearing of domestic animals, the main occupation which is farming runs from November to February. The people of Enugu are of Igbo ethnicity hence speaking Igbo language.

Malaria is endemic in Enugu state, and occurs all year round. Previous studies from the study area showed that patent medicine dealers (also known as patent medicine vendors) are the major source of treatment for malaria [2, 8, 24, 25]. These studies also found that chloroquine, SP and artesunate monotherapy were still widely available and used for the treatment of malaria.

### Study design

Qualitative methods including Focus Group Discussion (FGDs) and In-Depth Interviews (IDIs) were used to elicit information from community members and health workers, respectively. Pre-tested IDI and FGD topic guides were used to interview health workers and community members. Informed consent was obtained from the participants before the interviews and discussions were held. The IDIs and FGDs were conducted in the local language (Igbo). Information on demographic characteristics of all participants were collected using enrolment forms. For the purpose of anonymity, codes were assigned to participants in accordance to study site and classification. A trained team of 14 research assistants comprising of 7 interviewers and 7 note takers conducted the FGDs and IDIs.

### FGDs

Three villages were randomly selected in the two study sites of Enugu and Udi. Three FGDs were held in each village. A total of 18 FGDs were conducted involving 179 community members, making an average of 9 participants per group. Participants were purposively selected to provide a representative configuration of the population of community members frequently seeking care. Participants needed to be primary care givers within households and adult men and women aged 15 years and above. Village contact persons were identified by the research team in the two sites to help to mobilize and recruit their fellow villagers to participate in the FGDs. The contact persons were responsible for identifying and inviting eligible participants for the FGDs. The FGDs were conducted in quiet venues such as community halls. Topics explored in the FGDs included: places community members’ receive malaria treatment (in order to understand their current health seeking situation); their knowledge of how to handle childhood malaria (because of the vulnerability of children); different sources of information about malaria, malaria treatment and prevention and; finally, their views on the value of different types of information and effective strategies for delivering that information at the community level.

### IDIs

A total of 26 facilities (13 in each study site) were randomly selected from a sampling frame of public and private facilities based on a census of all facilities in the study areas that was undertaken as part of this study. Interviews were conducted with 26 purposively sampled health workers known to be actively involved in prescribing drugs across a range of public and private health facilities. These providers included nurses, midwives, community health extension workers, community health officers, pharmacy technicians, and patent medicine dealers. The IDI topics explored were: health workers knowledge of malaria and its appropriate prevention and treatment methods; sources of knowledge of malaria and its control; the importance of knowledge of malaria prevention and treatment; current methods for malaria communication; and improved channels for effective communication. IDIs were conducted in the workers' facilities.

### Data analysis

Recorded responses to the IDIs and FGDs were transcribed and translated into the English language by trained note takers. To promote the accuracy of the meaning-based translation, two field workers with a good knowledge of both English and Igbo languages back translated a sub sample of 20 % transcripts for both the FGDs and IDIs to compare how closely the two versions matched. Discrepancies were discussed and resolved. Coding of responses was completed by two sociologists.. Emerging issues were identified and categorized into themes as transcripts were being reviewed. The data were analyzed using NVIVO software version 8.

### Ethical aspects

Ethical approval was obtained from the University of Nigeria Teaching Hospital Ethical Committee (UNTH/CSA.329) and from the London School of Hygiene &Tropical Medicine (Approval 5429).

## Results

The average mean age of FGD participants was 41 years; 50 years for male and 33 years for female. Most participants were employed and had completed at least senior secondary education. The mean age of health workers in the IDIs was 38 years and they included both males (13 %) and females (87 %). Approximately 58 % of health workers were private medicine vendors and 42 % were from public health facilities and included nurses (8 %), nursing assistants (7 %), community health extension workers (CHEWs) (17 %) and other cadres (10 %). The majority of health workers had completed junior secondary school. The results below are grouped according to the key questions asked in the IDIs and FGDs: sources of malaria treatment; knowledge of malaria in children; sources of information on malaria treatment and prevention; and the relative importance and role of different sources of information.

### Sources of treatment for malaria by community members

Participants sought different treatments from a range of public and private health workers. Distance to a facility and availability of qualified staff and medicines were important factors influencing choice of provider.

*‘Okay, the hospital I go to is the government hospital. Either in my Local Government or State government i.e. government hospital or the clinic in my local government area ‘(P1, Enugu, FGD, Adult Men)*

Many visited patent medicine dealers (from 14 FGDs), health centres (from 13 FGDs) and private hospitals/clinics (from 11 FGDs) respectively to seek treatment for malaria.

‘*The place nearer to us where we receive treatment is the health centre’ (P8, Udi, Adult Women)*

*‘Me, usually..... Like last month I had malaria, there was no doctor so I went to a private clinic’ (P2, Udi, Adult Men)*

Some participants also visited traditional healers (2 FGDs) and pharmacies (2 FGDs) to seek malaria treatment. For these patients, seeking care was often a process of 'trial and error'; if conventional medicines were judged not to have worked, then herbal remedies were sought.

*Just as some the others have said, I use herbs. There is a herbal doctor in our place called “Oyi- umuaka. I go there for herbal treatment after orthodox drugs fail. He demonstrated with his hands and pointing to different places....... thanks’ [P9, Udi, Adult Men*].

*‘I go to pharmacy, buy Amalar and use it for treatment’ [P1, Enugu Adult Men]. 'I treat my own by going to a chemist, any drug they give me I take it; because I don't know about drugs. If I don't get better after drinking it, I go back to the chemist again, where they will ask me how the first drug they gave me reacted, I tell them, then they look for another type and give it to me. That's how I do my own because I cannot take Igbo traditional herbal medicine' [P4, Udi, Adult women].*

### Caregivers’ knowledge of a child with malaria

The majority of FGD participants identified fever and loss of appetite as the major symptoms of malaria in a child. Illustrative quotes from some of the FGD participants are given below.

‘*Once my child starts running fever and is always crying, then I know certainly is malaria that is disturbing him.’ [Baby shouts] (P8 FGD, Enugu, Primary Caregivers)*

‘*How I know is that my child will start having loss of appetite, and also gets weak always, I will now know it’s malaria, because I know the symptoms of malaria’.(P4, FGD Enugu, Primary Caregivers);*

Other caregivers (4 FGDs) stated that headaches and weakness of body were key signs of malaria as illustrated below.

*‘They will have headache as in baby will have headache ‘(P1, FGD, Primary Caregivers)*.

*‘My child has malaria at times it makes the baby weak; sometimes it makes the baby not to feed/eat well… (P4, FGD, Udi, Primary Caregivers)*

Contrary to the malaria treatment guidelines that recommend testing before treatment, most workers freely indicated that in their present practice, they diagnose malaria based on symptoms alone using a combination of the patient’s description of their symptoms and their own observations.

*‘Based on our standing orders we are basing on signs and symptoms, if somebody presents with febrile conditions, headache, dizziness then we suspect malaria… Then you administer malaria drug to the person…’ (IDI, Enugu Health centre, Nurse,)*

*‘What I learnt or trained for is much, malaria can come inform of fever, can make the person emaciate, sometimes if the colour of the person’s eyes and urine will change, when the patient explains these, you know it’s malaria’ (IDI, Udi, Drug Retailer).*

‘*Is it by physical observation ‘… if somebody just comes in and says I have weakness, headache, fever and all those symptoms of malaria, then you do not have any choice than to give the person malaria drug' (IDI, Enugu Pharmacy).*

*' Because even if I did not conduct any test maybe because of the signs and symptoms I have seen on the patients and I want to administer anti malaria treatment on the patient I will still use the same type of anti malaria … know that malaria has different stages since there is type 1, type 2 and type 3 it is when you test it, when the person has gone through test you now know whether it is in the second stage or first stage to know the drug to administer to get rid of that malaria because if the person does not undergo test example if we have laboratory here it would be very necessary to know the stage but since we do not have I treat according to symptoms and signs that we see' (IDI, Enugu, Health Centre, Nurse).*

### Sources and role of information on malaria prevention and treatment

Community members identified a range of press and print media including radio, television adverts and posters as key sources of information on malaria prevention and treatment as shown in the quotes below.

‘*I see posters and TV. In the Adverts, one man came and was advising people to keep their environment clean and tell the children to close windows and doors in the evenings. He said shutting the windows/door stops mosquitoes coming in’ (P8. Udi FGD Adult women].*

*' According to adverts on the television and radio, they will always advise that after taking a drug for two to three days, if symptoms continue, consult a doctor. So I wait and see the effect of the drugs I take for three to four days, if it heals me then I know that it is working, but if it doesn’t, I will consult a doctor’ [P7 Udi FGD Adult men].*

Focus group participants also relied on other community members, most commonly friends, neighbors and family, for information on malaria treatment irrespective of whether it was known to be effective or not.

*' It was from my neighbour who used a particular drug in time past so when my children took ill, she gave me the pack of the drug she used to treat her children and asked me to go and buy. So that was how I know artesunate' [P1 Enugu FGD primary care giver].*

*' How I recognized or know the artesunate is from my husband when he was sick, he went to lab test and he was given artesunate, so he drank it and he was okay. So now, when my Children is sick, I also give it to them'. [P1 Enugu FGD primary care giver].*

*‘… my friend recommended artesunate to me but the doctor prescribed Alaxin for me in the hospital … after my house help did a lab test and it was malaria, the doctor wrote Alaxin and other drugs, plus Halfan too and she finished drinking them, she quickly recovered because it was good on her. That’s how I know. So if malaria comes now and maybe I can’t go to hospital, I just go and get Alaxin or artesunate but since it is not the doctor that is giving it to me directly, I add blood tonic and paracetamol to give to my child. That’s how I learnt my own' [P2. Enugu. FGD primary care giver].*

Health workers typically sought information on how to treat malaria from their colleagues as shown in the quotes below.

*‘Moreover I have some medical doctor friends who I liaise with even when I have complicated issues I do phone them. Somebody like a certain doctor here, I do call him and he will direct me on how I will handle some complicated cases’ (IDI, Udi drug Retailer)*

‘*Ok of course yes some of the doctors that do treat, I use to observe the way that they treat (IDI, Enugu Health Centre Nurse)*

Health workers also relied on professional materials including the Nigerian standard treatment guidelines and publications from medical journals and books to help them diagnose and treat malaria patients.

*‘….Ok, from, like West African Post Graduate College of Pharmacy, we are talking about malaria, treatment procedures and all the rest of them, they even reference standard treatment Guideline. (IDI, Enugu Pharmacy)*

Health workers, especially pharmacists and PMDs, were open to information delivered through the media especially where it was produced by international organizations such as the World Health Organization.

*‘TV advert by WHO that people should go for ACT. Both combine to make one think of giving more of ACT and give less of the old’ (IDI, Enugu pharmacy*)

Health workers acknowledged training workshops organized by a range of groups such as the Ministry of Health and the Nigerian Agency for Food and Drug Administration and Control (NAFDAC) as another important source of up to date information on malaria treatment and diagnosis. It was also noted that in practice they did not always follow that training especially where less effective older antimalarials were more affordable for poorer patients.

*‘…….Yes it changed it because actually, we only relied on other malaria drugs like Amalar and Chloroquine but after the knowledge acquired from the training, we now accept this combination therapy as the best treatment of malaria’(IDI, Enugu Centre Nurse).*

*‘The training was more of acquainting us with the way malaria is common in this part of West Africa. So we were told that some of the drugs that most people are not responding to in terms of when they now have malaria… some of the drugs like this Chloroquine are fading out of fashion so the new drugs that are to be administered with dosages and the durations. And those things to look for before you now begin to suspect malaria on your patients. I also learnt through those workshops that we should not neglect malaria that it might lead to somebody’s death something like that so we have to make sure that we tackle it as quickly as possible so that it doesn’t get to that acute stage’ (IDI, Enugu Health Centre Nurse).*

*‘They taught us how to use the rapid malaria test, like the one I attended last year, they encouraged us to tell people to use this ACT that it is better and more powerful than other drugs because it is a combination' (IDI, Enugu Health Centre Nurse).*

*‘The workshop/training was ok because they emphasized mainly on the fact that the plasmodium parasite has become resistant to a lot of antimalaria and they emphasized the fact that combination products are better. Then they regarded the old ones as long acting and the new ones like Arthemeter and ehhh others as short acting and more effective but malaria parasite is getting resistant so combination works better. That is why emmm we want to give Coartem or any other combination of ACT instead of the old ones but if one does not have money to buy all these, the person does not have option than to buy the old one' (IDI, Enugu Pharmacy).*

Finally, health workers were confident that their personal experience and ‘on the job’ training provided them with much of the information necessary to effectively treat and diagnose their patients. Similarly, community members commonly felt that due to the high frequency of malaria episodes experienced, they too had considerable knowledge of the disease and could recognize symptoms.

*‘Nothing it’s just that when a patient enters here and complain on malaria from my experience I will know how to treat the sickness’ (IDI, Enugu Drug Retailer)*

*‘Well another thing that influences my practice is this because the bible says in an adage says that “experience is the best teacher” And as a health worker in the village, I confront many problems so from the additional experience I acquire from those complicated cases I handle, it helps in advancing my knowledge' (IDI, Udi, Drug Retailer).*

*' Before, I used to be very sick, always having malaria and the drugs I take from the chemist were not working so my friend..eemm. told me about Amalar, so I bought Amalar and recovered. Since then whenever I have malaria, I always buy Amalar’ [P5 FGD, Udi Adult women].*

*‘Like the sickness we suffer from since we were born, we got to know there is malaria, typhoid, when we go to hospital. We also got to know that malaria causes fever, cold, weakness of the body’ (P9. Udi FGD Adult women).*

### Importance and role of different sources of information for malaria prevention and treatment

Community members rely on information obtained from a variety of sources including television, radio and newspaper adverts on malaria, as well as malaria posters in hospitals and health centers to understand how malaria should be properly diagnosed and appropriate medicines used for treatment. Information from friends, family, neighbors and other community members was important in guiding decision making around preferred medicines for malaria treatment. Examples of the responses obtained from some FGD participants are provided below.

*' ………….I was using Chloroquine but it used to itch me but because I did not know which other drug to substitute it with I continued using it, but when I learnt about Amalar from an advert, I took Amalar (SP) but it did not heal me completely, before I saw Artesunate (monotherapy) advert on television which I then took and it healed me completely’ (P9. Enugu FGD Adult women).*

‘*I see posters and TV. In the Adverts, one man came and was advising people to keep their environment clean and tell the children to close windows and doors in the evenings. He said shutting the windows/door stops mosquitoes coming in’ (P8. Udi FGD Adult women].*

*‘Like the sickness we suffer from since we were born, we got to know there is malaria, typhoid, when we go to hospital. We also got to know that malaria causes fever, cold, weakness of the body’ (P9. Udi FGD Adult women).*

Health workers acknowledged participation in training workshops was of particular importance in understanding the changing landscape of malaria resistance, how to use RDTs for malaria and for understanding the new drug combinations for treating uncomplicated malaria and the complex dosage regimes that accompany these medicines.

*‘…….Yes it changed it because actually, we only relied on other malaria drugs like Amalar and Chloroquine but after the knowledge acquired from the training, we now accept this combination therapy as the best treatment of malaria’(IDI, Enugu Centre Nurse).*

*‘The training was more of acquainting us with the way malaria is common in this part of West Africa. So we were told that some of the drugs that most people are not responding to in terms of when they now have malaria… some of the drugs like this Chloroquine are fading out of fashion so the new drugs that are to be administered with dosages and the durations. And those things to look for before you now begin to suspect malaria on your patients. I also learnt through those workshops that we should not neglect malaria that it might lead to somebody’s death something like that so we have to make sure that we tackle it as quickly as possible so that it doesn’t get to that acute stage’ (IDI, Enugu Health Centre Nurse).*

‘*They taught us how to use the rapid malaria test, like the one I attended last year, they encouraged us to tell people to use this ACT that it is better and more powerful than other drugs because it is a combination ‘(IDI, Enugu Health Centre Nurse).*

### Suggested channels for effective communication on malaria prevention and treatment: community members and providers’ perspectives

Across the 18 FGDs held for community members, participants suggested the use of various channels for communicating information about malaria prevention and treatment to members of the public. A number of routine channels for communication were identified including the dissemination of written information through health centres and hospitals as well as adverts and messages through the media, especially radio, television, newspaper and magazines. Other suggestions included holding public meetings in villages, market places, and schools. Church announcements were also flagged as an alternative avenue for communicating messages about appropriate malaria treatment and prevention. It was emphasized that a multi-pronged approach was required since one source may not meet the needs of all members of the community especially those those from different geographic areas or from different socio-economic groups.

*‘The way to circulate information is to print it on paper and give to churches because it is churches that people use this day. Then, you people will come to churches and talk to the congregation’ (Demonstrates using his hand and legs)' [P2. Udi FGD Adult men].*

*‘The best is the radio because not everybody will get up to go and watch TV in somebody’s house. So a lot of people have radio than TV. Like myself I will not go to somebody’s house to watch TV advertisement on health while radio is in my house. Radio is better’ [P2. Enugu FGD Adult women].*

Furthermore, approximately half of the health workers interviewed emphasized the importance of communicating through existing channels such as government training programmes, support visits to the health workers and through health workers' unions. Other complementary means of communication included the use of radio jingles, through TV adverts, advert by drug manufacturers, using handbills, posters and bill boards.

*‘The way I prefer is for you to call us, because if say it is on radio, some may not have time to listen to the radio. In the morning the person takes his bath and leaves the house and goes to the shop and stays. So the best way is for you to call us and tell everybody to come with a biro and you teach us do it this way and that way, just doing it face to face, for me I like it best’ (IDI, Udi Drug Retailer).*

*I feel that it is through training’ (IDI, Udi Drug Retailer).*

*‘Em, I think this system or technique you now use will be very helpful to us Nigerians generally, now like today I know you have my phone number, and here (touching the information sheet given to him) I know you have your phone numbers here and I believe that this is an easy form of communication and if we ourselves are able to liaise with people like you, it will help in enhancing our profession and work and it will improve our understanding about the business’(IDI, Udi, Drug Retailer) .*

## Discussion

Strategies for effective malaria treatment will be enhanced if both consumers and providers have optimal information to help them understand appropriate methods for malaria prevention and treatment. Health workers and community members are recognized as important participants, consumers and implementers of malaria control interventions. Effective communication between these groups is critical in order to bring about sustained improvements in malaria treatment and prevention. More broadly, it can also encourage individuals at the community level to participate in activities related to their own development [26] Understanding and appropriately utilizing the variety of existing sources of information is a critical first step in the design and implementation of communication and behavior change interventions for malaria control.

This study showed that harnessing multiple sources of information to empower consumers is important. In many situations, patients rely on a health workers' judgment to meet their health needs. But it is also common for patients to rely on inaccurate information on malaria treatment provided by friends or relatives. Hence, unless health workers and community members are informed through appropriate channels about the most effective tools for preventing and treating malaria, their welfare will suffer [13]. Other studies found that health care workers provided inappropriate treatment due to lack of awareness of appropriate malaria treatment guidelines [25, 27–31].

This study showed that the major sources or channels through which information was transmitted were the mass media, churches, schools and village announcements, through opinion leaders and through journals and workshops for health workers. This is in line with strategies identified in the communication strategic framework developed by the government of Nigeria to be used for malaria prevention and treatment awareness creation [1]. However, despite the different sources of malaria information reported in this study, some researchers have argued that the use of television, radio or newspaper may not address the major underlying problems in malaria control in many developing countries which are mainly caused by inequality in wealth distribution and inequitable access to technology among different socio-economic class [26].

The findings from this study also suggest that community involvement in the dissemination of information on malaria control has the potential to improve community awareness, attitudes and practices in relation to malaria control. This is in line with other studies which show that improving information and communication at the community level improves participatory development, a method that improves use of local medium, knowledge, information and decisions to manage an intervention [32–35]. Village announcers and opinion leaders were deemed acceptable sources of malaria information and are a potential low-cost strategy for community-level dissemination of information for the control of malaria. The community opinion leaders could be mobilized and empowered with appropriate and regular information on malaria for them to serve as change agents for improving the treatment and prevention of malaria at the community level. This approach has been advocated in a range of malaria endemic settings [13, 18, 33].

Another inference from the findings of this study is that for malaria control programmes to achieve an appreciable positive change in treatment seeking behaviour of community members, information on effective malaria treatment should also be provided using printed materials and the mass media. The suggested print materials included pamphlets, posters and church bulletins. Media sources included radio, newspapers, magazines and television. Using the mass media as a channel for informing the public about a positive behaviour has been shown to create demand for services, reinforce advice, motivate and remind people of their previous knowledge [13, 20, 36–38].

Effective training of health workers has been argued to be useful in empowering them with information to understand malaria treatment guidelines and provide opportunities for them to exchange ideas with their colleagues as found in other studies [13, 20, 37, 39]. Some health workers in this study expressed a need for further training so as to improve their current practice and skills and in turn promote the appropriate treatment of malaria. Other studies have reported that the demand by some health workers for further training is also motivated by a desire or need for increased salary or payment of per diems while attending training [34]. However, this is not always true as many health workers may be motivated by the fact that their knowledge and capacity to properly treat malaria will be enhanced by such capacity development activities.

A limitation of this study was the fact that due to time constraints, we were unable to screen FGD participants before undertaking the discussions so as to ensure participants do not come from one particular household or family. In 3 FGDs, some participants were found to be from the same household, but they all met the eligibility criteria of being a care giver. Also, the fact that the interviews were undertaken in the local language and then translated into English could potentially lead to a loss of meaning [39]. Finally, it was beyond the scope of this study to examine the effect of level of education on knowledge of malaria and the relative effects of different channels of communication on improving malaria knowledge, provision and use of appropriate prevention and treatment tools for the disease control.

In conclusion, there are many different channels through which information on malaria treatment and prevention is currently delivered to healthcare consumers and providers that could be harnessed to significantly improve the control of malaria. For increased effectiveness and sustainability, systematic and deliberate efforts should be made by malaria control program managers and other actors to involve communities and health workers in the design and implementation of information, communication and behavior change interventions that are targeted at improving the treatment and prevention of malaria. Such an approach will increase the acceptability, trust and use of the acquired knowledge by all actors for improved malaria control.
